# Insights into dendritic cell maturation during infection with application of advanced imaging techniques

**DOI:** 10.3389/fcimb.2023.1140765

**Published:** 2023-03-02

**Authors:** Qi Xiao, Yuxian Xia

**Affiliations:** ^1^ Genetic Engineering Research Center, School of Life Sciences, Chongqing University, Chongqing, China; ^2^ Chongqing Engineering Research Center for Fungal Insecticide, Chongqing, China; ^3^ Key Laboratory of Gene Function and Regulation Technologies Under Chongqing Municipal Education Commission, Chongqing, China

**Keywords:** dendritic cells (DCs), antigen presentation, maturation, migration, imaging, DC-T cell interaction

## Abstract

Dendritic cells (DCs) are crucial for the initiation and regulation of adaptive immune responses. When encountering immune stimulus such as bacterial and viral infection, parasite invasion and dead cell debris, DCs capture antigens, mature, acquire immunostimulatory activity and transmit the immune information to naïve T cells. Then activated cytotoxic CD8^+^ T cells directly kill the infected cells, while CD4^+^ T helper cells release cytokines to aid the activity of other immune cells, and help B cells produce antibodies. Thus, detailed insights into the DC maturation process are necessary for us to understand the working principle of immune system, and develop new medical treatments for infection, cancer and autoimmune disease. This review summarizes the DC maturation process, including environment sensing and antigen sampling by resting DCs, antigen processing and presentation on the cell surface, DC migration, DC-T cell interaction and T cell activation. Application of advanced imaging modalities allows visualization of subcellular and molecular processes in a super-high resolution. The spatiotemporal tracking of DCs position and migration reveals dynamics of DC behavior during infection, shedding novel lights on DC biology.

## 1 Introduction

As the most efficient antigen-presenting cells (APCs), Dendritic cells (DCs) keep sensing environmental stimulus such as infection and injury, recognize and take up pathogenic antigens. Contact and uptake of microbial components trigger the morphological, phenotypic and functional changes of DCs. This process is known as DC maturation (Dalod et al., 2014; Cabeza-Cabrerizo et al., 2021). Mature DCs acquire the capacity to migrate and transport the processed antigens from peripheral tissues to lymphoid organs, convey the information to other immune cells and prime the naïve T cells. Meanwhile, DCs also regulate the innate immune response through recruiting neutrophils and inducing the proliferation and cytotoxicity of natural killer cells (NKs) (Ferlazzo et al., 2004; Granucci et al., 2004). Therefore, DCs form a bridge connecting innate and adaptive immunity.

In the past twenty years, the rapid development of optic technologies has revolutionized the way we study the immune cells and immune response. Advanced imaging techniques and illumination devices such as intravital imaging with multiphoton microscopy, light-sheet microscopy, atomic force microscopy, and super-resolution microscopy (Vyas, 2012; Oreopoulos et al., 2014; Tam and Merino, 2015; Yildiz and Vale, 2015; Schiessl and Castrop, 2016; Girkin and Carvalho, 2018), enable us to identify the precise localization of distinct cell subtypes, track the cell migration through time-lapse imaging, monitor the *in vivo* cell contacts in single cell resolution, and explore the molecular mechanisms that underlie these cell behaviors and interactions. For example, intravital imaging observes dynamic organisms in live, intact animals, providing continuous and simultaneous insights in an intact organism with complete interactions. Compared with confocal microscopy, multiphoton microscopy uses near simultaneous absorption of multiple long wavelength photons, which guanrantees the excitation at the focal plane; Atomic force microscopy is a powerful imaging and analysis tool for obtaining high-resolution nanoscale images of cell surface; Super resolution microscopy overcome the diffraction limited resolution of light microscope and obtain high spatial resolution. Stochastic optical reconstruction microscopy (STORM) and stimulated emission depletion microscopy (STED) are the most popular super-resolution techniques which are used to reveal the nanoscale organization of different structures in cells. Application and advantage of these imaging techniques are summarized in **Table 1**. These imaging modalities permit direct observation of DC maturation process with good spatiotemporal resolution, making a great contribution to our understanding of the antigen processing, DC migration, T cell priming and immune response initiation. Therefore, this review aims to provide an overview of the innovative research with advanced imaging techniques that analyze the behavior and function of DCs after infection, and the application of optical technology in studying the fundamental immunology questions.

**Table 1:** Characteristics and application of advanced imaging techniques.

	Characteristics	Advantages	Limitations	Application
Intravital imaging with multi-photon microscopy	Observe dynamic organisms in live animals in a spatiotemporal manner; Simultaneous absorption of multiple photons in multiphoton microscopy guarantees the excitation at the focal plane	Live animal imaging; No out-of-focus light; Less damaging to the tissue; Extended observing time and deep penetration within tissues	Multiphoton microscopy provides a lower resolution than confocal microscopy	Many research areas, such as immunology, tumor biology and neurobiology.
Atomic force microscopy (AFM)	Surface analysis tool for obtaining high- resolution nanoscale images. Offer information on physical properties (size, morphology, surface texture and roughness); Measure forces (adhesion strength, magnetic forces and mechanical properties)	Compared with scanning electron microscopy, AFM does not require special treatments (metal/carbon coatings); Work in both air and vacuum; Provide a true 3-D surface profile	Lateral resolution is not sufficient for detailed structural studies.	Biochemistry and biophysics applications (structure of biological molecules, cellular components), materials science and nanotechnology applications
Spinning disk confocal microscopy (SDCM)	Use a rotating disk with thousands of pinhole apertures, thousands of emission light scan the specimen simultaneously. Images are taken at a focal plane and out- of-focus lights are discarded	Compared with laser scanning confocal microscopy, SDCM has lower light levels; More efficient fluorescent detection; More accurate cell physiology	Inability to adjust the pinhole size to alter the optical sectioning strength; Pinhole cross-talk effect creates background signals.	Imaging fast dynamic processes and live specimens
Super resolution microscopy (SRM)	STORM reconstructs super-resolution image by combining the high-accuracy localization information of individual photo-switchable fluorophores.	Standard organic fluorescent dyes; Simple instrument and highest resolution on an optical imaging system in biological application.	Extensive post- acquisition image processing for image reconstruction	Detect molecular interaction and dynamics, visualize nanoscale structures with optical techniques, study
	STED switches off the fluorophores out of the diffraction limited excitation focus. Fluorescence from the excited dye molecules in the center of the focus is detected and form the high-resolution images.	Simple and fast acquisition process; Deep tissue imaging; Fast acquisition without the need for additional data processing	Increased photobleaching	fundamentals of biology through single molecule fluorescence.
Light sheet microscopy (LSM)	Thin sheet of light orthogonal to the detection plane scans plane by plane, collects the fluorescence signal of the observed region to reconstruct 3D images.	Compared with confocal microscopy, LSM reduces photobleaching effects; High signal-to-noise ratio and fast scanning rate, useful to image large scale specimens.	Transparent sample required.	Embryonic development, whole brain neural activity, immune cell interaction and motility.
Total internal refection fluorescence microscopy (TIRFM)	Use laser light at an angle to only excite fluorophores that are located near the cell membrane interface, and restrict the zone of observation to the plasma membrane or just beneath it.	Offers a much reduced background fluorescence	Total internal refection occurs only at the interface, more suitable for generating optical sections of 2D but not 3D images	Intracellular cargo transport, actin dynamics near the plasma membrane, and focal adhesions in living cells.

## 2 DC subsets

**Figure 1:**
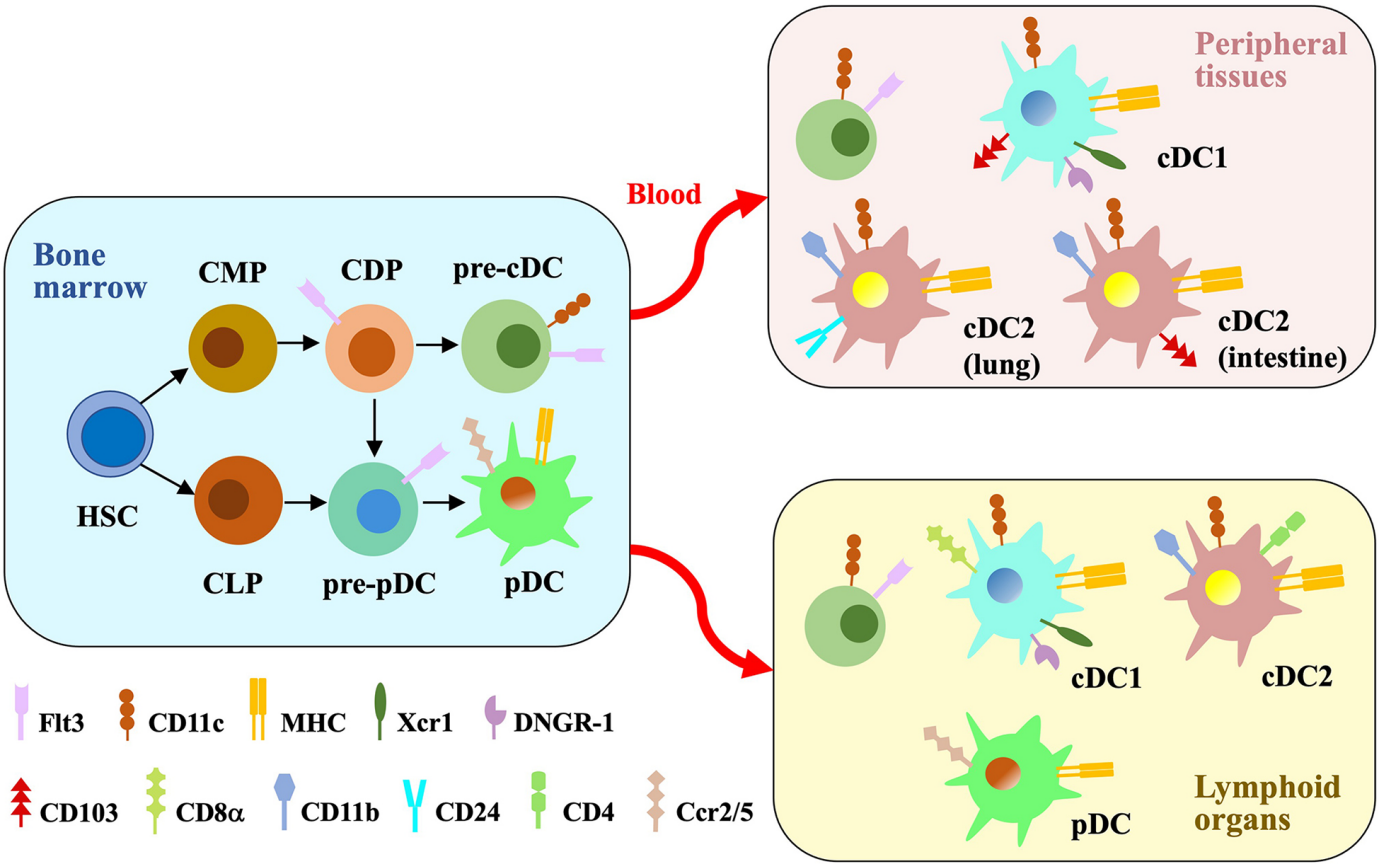
Major DC subsets. In the bone marrow, hematopoietic stem cells (HSCs) differentiate into common myeloid progenitors (CMPs) and common lymphoid progenitors (CLPs). CMPs generate DC-fate restricted progenitors called common DC progenitors (CDPs), which are the common progenitors of pre-cDCs and pre-pDCs. Pre-pDCs are also produced from CLPs. Flt3 binding to the ligand induce the differentiation process. Pre-cDCs are released into the blood to reside in peripheral tissues and lymphoid organs, where they differentiate into cDC1s and cDC2s. pDCs get mature in the bone marrow and then migrate to peripheral lymphoid organs through blood. cDCs and pDCs express different cell surface proteins which distinguish their subsets.

DCs, named for their dendritic-shape processes, were first discovered 50 years ago (Steinman and Cohn, 1973). The ontogeny studies and gene expression profiling divide DCs into two main subsets: conventional (or classical) DCs (cDCs) and plasmacytoid DCs (pDCs) (Cabeza-Cabrerizo et al., 2021). cDCs, developing from myeloid lineage, capture and present antigens to naïve T cells to induce immune responses. (Anderson et al., 2018). pDCs are specialized for producing large amounts of type I interferons (IFN-1) in response to viral infection (Reizis, 2019). Initially, researchers considered that cDCs and pDCs are generated from common progenitor cells (Naik et al., 2007; Onai et al., 2007). Recent studies using single-cell RNAseq together with *in vivo* fate mapping found that pDCs also developed from lymphoid progenitor cells (Rodrigues et al., 2018; Dress et al., 2019) (**Figure 1**). A third subpopulation, monocyte-derived DCs (moDCs) are ontogenetically distinct from cDCs (Cabeza-Cabrerizo et al., 2021). Previous study observed antigen-presentation functions of moDCs (Hohl et al., 2009; Langlet et al., 2012). However, it could be due to a mixture of monocyte-derived cells with a subpopulation of inflammatory cDCs. After removing cDCs, moDCs were unable to present antigens to T cells (Bosteels et al., 2020). The exact function of moDC *in vivo* is still unclear. They might be less migratory than cDCs and rather function at the infection site, produce higher level of inflammatory cytokines and chemokines, and orchestrate inflammatory response locally (Guilliams and van de Laar, 2015; Backer et al., 2023). In this review, we mainly focus our discussion on the response, behavior and function of cDCs and pDCs after the pathogen invasion.

### 2.1 cDC

cDCs originate from hematopoietic stem cells (HSCs) in the bone marrow. HSCs generate intermediate, DC-fate restricted progenitors called common DC progenitors (CDPs), which differentiate into pre-cDCs depending on the growth factor FMS-like tyrosine kinase 3 (Flt3) binding to its ligand. Pre-cDCs migrate out of the bone marrow, travel through the bloodstream and differentiate into cDCs in the lymphoid and peripheral organs such as spleen, lymph nodes, intestine and lungs. Differentiated cDCs in both lymphoid and peripheral organs highly express major histocompatibility complex (MHC) molecules for antigen presentation, and cluster of differentiation (CD)11c. cDCs contain two subgroups, cDC1 and cDC2 (Schlitzer et al., 2015). They express different markers and rely on distinct transcriptional factors for development (Nutt and Chopin, 2020).

#### 2.1.1 cDC1

All cDC1s express the chemokine receptor Xcr1 and the C-type lectin receptos DNGR-1 (Poulin et al., 2012; Guilliams et al., 2016). Additionally, cDC1s in the lymphoid organs express CD8α (Vremec et al., 1992), while those in the peripheral tissues express CD103 (Edelson et al., 2010). cDC1 differentiation requires transcription factors Irf8 (Schiavoni et al., 2002), Batf3 (Hildner et al., 2008), Id2 (Hacker et al., 2003) and Nfil3 (Kashiwada et al., 2011). cDC1s are specialized for efficently presenting exogenous antigens to CD8+ T cells (Hildner et al., 2008) and promoting cytotoxicity T cell immunity. This process requests MHC-I molecules for antigen loading, and is called cross-presentation (Alloatti et al., 2016; Embgenbroich and Burgdorf, 2018). We will discuss it in detail in the antigen processing section. cDC1s also play an important role in early priming of CD4+ T cells in the context of tumor-derived antigens (Ferris et al., 2020). In addition, cDC1s produce interleukin (IL-)12 to control microbial infection (e.g. Toxoplasma gondii) (Mashayekhi et al., 2011; Poncet et al., 2019) and promote T helper (Th) 1 cell differentiation (Maldonado-López et al., 1999; Zhou et al., 2018; Yin et al., 2021).

Batf3 plays a highly important role in regulating cDC1 development. Batf3 is specifically expressed in cDCs, with low or absent expression in other immune cells (Hildner et al., 2008). Even Batf3 is expressed by both cDC1 and cDC2, lack of Batf3 expression only affects the development of the CD8α^+^ cDC1 (Murphy et al., 2013). Batf3 deficiency led to lack of antigen cross-presenting cDC1s, impaired cytotoxic T lymphocyte (CTL) response against viral infection, and high susceptibility to syngeneic tumors and intracellular parasite infection (Hildner et al., 2008; Mashayekhi et al., 2011). In addition, Batf3 maintained autoactivation of Irf8, which is crucial for the differentiation of pre-cDC1 to fully developed CD8α^+^ cDC1 (Grajales-Reyes et al., 2015).

#### 2.1.2 cDC2

Most cDC2s express high level of CD11b. cDC2s in lymphoid organs (lymph node, spleen) also express CD4 (Vremec et al., 2000), while those in peripheral organs (lung and intestine) express CD24 and CD103, respectively (Cabeza-Cabrerizo et al., 2021). cDC2 differentiation requires Relb (Burkly et al., 1995) and Irf4 (Tamura et al., 2005). cDC2s could be further split into two subpopulations. Initially, two subsets of cDC2s are distinguished based on the expression of Notch2 and KLF4 (Nutt and Chopin, 2020). Notch2-dependent cDC2 plays a key role in responding *Citrobacter* infection (Satpathy et al., 2013); KLF4^+^ cDC2s are crucial for promoting Th2 responses (Tussiwand et al., 2015). Recent study analyzed the cell heterogeneity using single-cell RNAseq and divided cDC2 into two new subsets: Tbx21 (T-BET)^+^ cDC2a and Rorc (RORγT)^+^ cDC2b (Brown et al., 2019). cDC2s recognize and present exogenous antigens to CD4^+^ T cells, support differentiation of Th2 and Th17 cell and promote T helper cell-mediated immune responses. (Gao et al., 2013; Persson et al., 2013; Schlitzer et al., 2013; Williams et al., 2013).

### 2.2 pDC

pDCs are continuously generated in the bone marrow from both myeloid CDPs and IL-7R^+^ lymphoid progenitor cells, forming a heterogenous population (Swiecki and Colonna, 2015; Rodrigues et al., 2018). Both pDCs express high amounts of transcriptional factor E2-2 and Irf8 that are critical for their development (Cisse et al., 2008; Rodrigues et al., 2018). Two subgroups of pDCs exhibit similar morphology and phenotype, however, single-cell RNAseq analysis revealed that they are obviously heterogenous. The CDP-derived pDCs are similar to cDCs, expressing high level of myeloid-related genes such as Zbtb46 and Klf4. Although both pDCs produce large amounts of type I IFNs in response to viral infection, only myeloid-derived pDCs are able to process and present antigens (Swiecki and Colonna, 2015; Rodrigues et al., 2018). When pDCs get mature in the bone marrow, they migrate to and reside in the peripheral lymphoid organs (Reizis, 2019). Mature pDCs express high level of chemokine receptors Ccr2 and Ccr5 that are necessary for their migration to the spleen and lymph nodes. pDCs also express low levels of MHC-II which can be upregulated upon activation (Sawai et al., 2013; Reizis, 2019).

## 3 DC maturation

In response to environmental stimulus such as infection and injury, the resting DCs in lymphoid or peripheral organs increase the expression level of MHC molecules and co-stimulatory molecules, e.g. CD80, CD40, CD86. DCs then become competent to process and present specific antigens to naïve T cells, and prime the immune responses. This process, named functional maturation, is the key features of DC biology. Unlike the traditional studies using fixed samples with very limited information on dynamic biological processes, advanced imaging techniques allow us to observe “real-time” cell behavior *in situ*, which greatly improvs our understanding of the spatiotemporal relationship between pathogens and DCs, DCs and other immune cells. The cutting-edge imaging techniques together with transgenic reporter mice which label a specific DC subtype with unique fluorescent markers (Lindquist et al., 2004; Satpathy et al., 2012; Yamazaki et al., 2013; Kitano et al., 2016; Brown et al., 2019), provide new insights on DC biology and challenge many dogmas. Here we summarize the key findings of DC maturation, including antigen-sampling, DC morphological change, peptide-MHC complex formation, DC migration, and DC-T cell interaction. DC maturation is also crucial for maintaining immune tolerance, which has been reviewed in other literatures (Iberg et al., 2017; Waisman et al., 2017) and will not be discussed here. This review focuses on mouse studies, but many findings and concepts can be applied to understand the immune response and immune system disorders in human.

### 3.1 Sensing receptors

DCs detect microbial components and other threat factors during infection through a diverse repertoire of immune receptors named pattern recognition receptors (PRRs) that recognize different pathogen-associated molecular patterns (PAMPs) in microorganisms. PRRs include toll-like receptors (TLRs), C-type lectin receptors (CLRs) that detect damage-associated molecular patterns (DAMPs) exposed by damaged cells, nucleotide oligomerization domain (NOD)-like receptors (NLRs) and retinoic acid-inducible gene-I-like receptors (RLRs) (Dalod et al., 2014; Cabeza-Cabrerizo et al., 2021; Li and Wu, 2021). Diversified surface and intracellular PRRs endow DCs with the capacity to detect multiple types of antigens: proteins, carbohydrates, lipids and nucleic acids (Takeuchi and Akira, 2010).

#### 3.1.1 PRR in DC subsets

The TLR family is one of the most important PRR families. They sense pathogens outside the cell or in the intracellular organelles. TLRs are expressed on the cell surface (e.g. TLR1, TLR2, TLR4, and TLR6) or in intracellular endosomes (e.g. TLR3, TLR7 and TLR9) (Segura et al., 2010). Different DC subsets express different TLR combinations. cDC1s express high level of TLR3 and TLR11. Endosome localized TLR3 is specialized for sensing and binding viral double-strand RNA (dsRNA) (Alexopoulou et al., 2001; Mielcarska et al., 2020), which is crucial for cross-priming of CD8^+^ T cells against virus infection (Schulz et al., 2005; Davey et al., 2010); Endo-lysosomal localized TLR11 recognizes and binds to profilin protein produced by *Toxoplasma gondii*, which initiates IL-12 production and induces the immune response against the parasite invasion (Yarovinsky et al., 2005). TLR12 cooperates with TLR11 to induce the host defense against parasites (Raetz et al., 2013). cDC1s also express DNGR-1, a plasma membrane localized CLR (Cueto et al., 2020), which recognizes the cytoskeletal components (e.g. spectrin-actin complex) from dead cells after the cell membrane is ruptured (Zhang et al., 2012). In addition, DNGR-1 binding to ligand actively induces phagosome rupture and dead cell-associated antigens release. These antigens bind to MHC class I molecules in the cytosol and are presented to CD8^+^ T cells, inducing cytotoxic T cell response against damaged cells. Three-dimensional (3D) correlative light and electron microscopy observed the ultrastructure of phagosomes, clearly showed a large hole in the phagosomal membrane for luminal contents to escape into the cytosol (Canton et al., 2021). Thus, DNGR-1 is necessary to prime CD8^+^ T cell in anti-viral or tumor immunity.

Different from cDC1s that efficiently detect intracellular antigens (including the exogenous antigens processed in the endocytic or phagocytic pathway), cDC2s detect extracellular pathogens. Mass spectrometry (MS)-based proteomics and transcriptomic analysis revealed multiple PRRs expressed in cDC2s, such as TLR1, TLR5, TLR6 and Clec4A (Crozat et al., 2009; Segura et al., 2010). cDC2s in the intestine detect the flagellin of pathogenic bacteria *Salmonella typhimurium* through TLR5, produce cytokine IL-6 and induce innate immune responses (Uematsu et al., 2006). Intestine cDC2s also promote Th17 cell development after TLR5 stimulation by commensal bacteria flagellin (Liu et al., 2016). In addition, cDC2 express Dectin-1 at plasma membrane to recognize β-glucan, the major cell wall component of fungi (Saijo and Iwakura, 2011; Ito et al., 2017). Activation of Dectin-1 promotes production of ROS from cDCs to kill fungi. These cDCs also secrete cytokines to recruit neutrophils for fungi clearing (Saijo and Iwakura, 2011).

pDCs specifically express endosomal TLR7 and TLR9 to sense nuclei acids. TLR7 recognizes virus and self-RNA, while TLR9 detects virus and self-DNA. Detection and internalization of virus nuclei acids activate TLR downstream signaling pathways, promote secretion of type I IFNs and other chemokines and cytokines, and induce the immune response against the virus (Swiecki and Colonna, 2015). pDCs also recognize profilin protein of *Toxoplasma gondii* through TLR12, similar to the response of cDC1. After detecting the parasites, pDCs produce IL-12 and IFN-α to activate natural killer (NK) cells, which release IFN-γ to reduce the parasite infection (Koblansky et al., 2013).

The different subcellular localizations of PRRs cover the invasion routes of different pathogens. Bacterial antigens are recognized by TLRs localized at the plasma membrane. If the bacteria escape and enter the cytoplasm, the intracellular NLRs (NOD1 and NOD2) are responsible for recognizing the components of bacterial cell wall. Fungi are specifically identified by plasma-membrane localized CLR Dectin-1 and Dectin-2 through β-Glucan and α-Mannan, both of which constitute the fungal cell wall. Virus release nuclei acids inside the cell, which are recognized by endosome-localized TLRs (TLR3, 7 and 9) or RLRs in the cytoplasm. Profilin protein of parasite are recognized by specialized PRRs TLR11 and TLR12 (Li and Wu, 2021). Thus, different pathogens are recognized by different PRRs, which induce special signaling pathway and cytokine production in the following DC maturation process.

#### 3.1.2 PRR signaling pathways

Downstream signaling pathways of PRRs determine cytokines and interferons production, T cell activation and immune response induction. Different TLRs first bind to one of adaptor molecules, MYD88, TRIF, TRAM and TIRAP, through their cytoplasmic Toll/IL-1R homology (TIR)-domain to activate downstream signaling pathways. There are three main signaling pathways: nuclear factor-κB (NF-κB), mitogen-activated protein kinase (MAPK) and interferon regulatory factors (IFRs). Most TLRs recruit MYD88 adaptor and then activate NF-κB or MAPK pathways for cytokine production and T helper cell polarization. TLR3 recognizing virus RNA interact with a different adaptor TRIF, and activate IRF signaling pathway, which induce the expression of IFN-1. RIG-1, a RLR that recognizes virus in the cytoplasm also activates IRF pathway to induce antiviral immune response. Therefore, bacterial invasion usually activates the transcription of NF-κB or MAPK pathway, and promotes the expression of pro-inflammatory cytokines, such as tumor necrosis factor (TNF), IL-6 and chemokines. Fungi and parasites infection also activate NF-κB and MAPK pathway based on the types of PRRs that recognize these pathogens. Differently, viral infection induces the synthesis of IFN-1 through IRF pathway, which promotes the expression of IFN-α and IFN-β to exert antiviral effects. In summary, different pathogen infections specifically induce the expression of immune effectors that target these pathogens (Dalod et al., 2014).

### 3.2 Antigen sampling

#### 3.2.1 Lung

Steady-state DCs residing in the lymphoid and peripheral organs keep monitoring the environmental changes by extending and retracting dendrites. In barrier organs such as the lung and intestine, DCs located at mucosal surfaces continuously sample antigens in the lumen. In the early time, researchers studied the change of cell morphology and phenotype through fixed tissues, which provided limited information of the dynamic DC behavior. Along with the development of optical technology, real-time imaging by intravital two photon microscopy allows researchers to investigate the cell behavior in a spatiotemporal resolution. Initial immunofluorescence staining on the fixed rat tracheal tissue showed that DCs beneath the epithelial cells projected their extensions to the apical surface when stimulated with antigens. This study indicated that DCs sensed the airborne pathogens in the conducting airways through their extensions (Jahnsen et al., 2006). Later study deeply explored the DC behavior in the steady-state or after airway inflammation by two-photon live imaging on viable lung slices and intravital lung. The study found a small fraction of motile airway-adjacent DCs protruded dendrites toward the epithelial cells. However, these dendrites did not pass the outermost epithelium or into the lumen. On the contrary, alveolar DCs with little motility kept extending and retracting dendrites across epithelial barriers and into airspace. But the number and the surface area of extended dendrites had no difference between the steady- and allergen challenged-state. Thus, alveolar but not airway DCs are responsible for antigen surveillance in the lung (Thornton et al., 2012).

#### 3.2.2 Intestine

DCs in the intestine open the tight junctions between the epithelial cells, protrude the dendrites outside the epithelium, sample and take up the pathogens (Rescigno et al., 2001). Intravital two-photon imaging further revealed that DCs in the proximal jejunum of the small bowel had much more trans-epithelial extensions into the lumen than those in the terminal ileum. But the microbial stimuli could significantly increase the extension number and frequency of DCs in the terminal ileum for active antigen sampling (Chieppa et al., 2006). These studies showed that DCs locations represented their different capacity of antigen sampling by transepithelial extension. Another study found that bacterial challenge on the luminal surface of intestine quickly recruited CD103^+^ DCs from the lamina propria to the epithelium. Then the intraepithelial DCs sent dendrites across the epithelial cells into the lumen to capture the bacteria. These DCs were also able to sample and capture soluble antigens in the intestinal lumen through their extended dendrites (Farache et al., 2013). Similarly, monocytic-origin DCs in the kidney sampled and captured the blood-borne pathogen through extending dendrites across endothelial layer and into renal cortical capillaries (Yatim et al., 2016). This behavior is usually mediated through TLR signaling pathway in epithelial cells (Chieppa et al., 2006; Farache et al., 2013). In summary, part of steady-state DCs actively sample the environmental antigens through dendrites extension. When infection happens, more DCs are recruited and capture the pathogenic antigens. However, whether the epithelial cells or surveillant DCs spread the recruiting signals and the detailed signal transduction pathway between cells are still not clear.

#### 3.2.3 Lymph node

The lymph node-resident DCs directly scan antigens that freely drain in the lymph. CD11b^+^ cDC2s positioned in subcapsular sinus of lymph node sample antigens in lymph fluids by extending highly motile dendritic processes into luminal space, directly catch and uptake the lymph-born microbial pathogens and vaccination-derived particulates (Gerner et al., 2015). Antigen processing and presentation by lymph node-resident cDCs initiate rapid CD4^+^ and CD8^+^ T cell activation independent of migratory peripheral DCs. Resident DCs infected by freely draining virions interacted with CD8^+^ T cells and stimulated anti-virus immune response rapidly. They also captured viral antigens by C-type lectin receptor SIGN-R1 and presented the antigens to CD4^+^ T cells (Hickman et al., 2008; Gonzalez et al., 2010). When parasites *Plasmodium* infected skin, the mobile sporozoites accessed skin-draining lymph nodes and directly delivered antigens to the resident CD8α^+^ cDC1s. Multi-photon intravital imaging revealed that the lymph node-resident DCs, instead of skin-derived DCs, were necessary and sufficient for antigen acquisition and activation of CD8^+^ T cell against malaria infection (Radtke et al., 2015). These studies indicate that when pathogenic antigens freely enter the afferent lymph, the lymph node-resident DCs could induce early T cell activation and initiate adaptive immunity independent of migratory DCs from peripheral tissues.

### 3.3 Antigen processing

**Figure 2:**
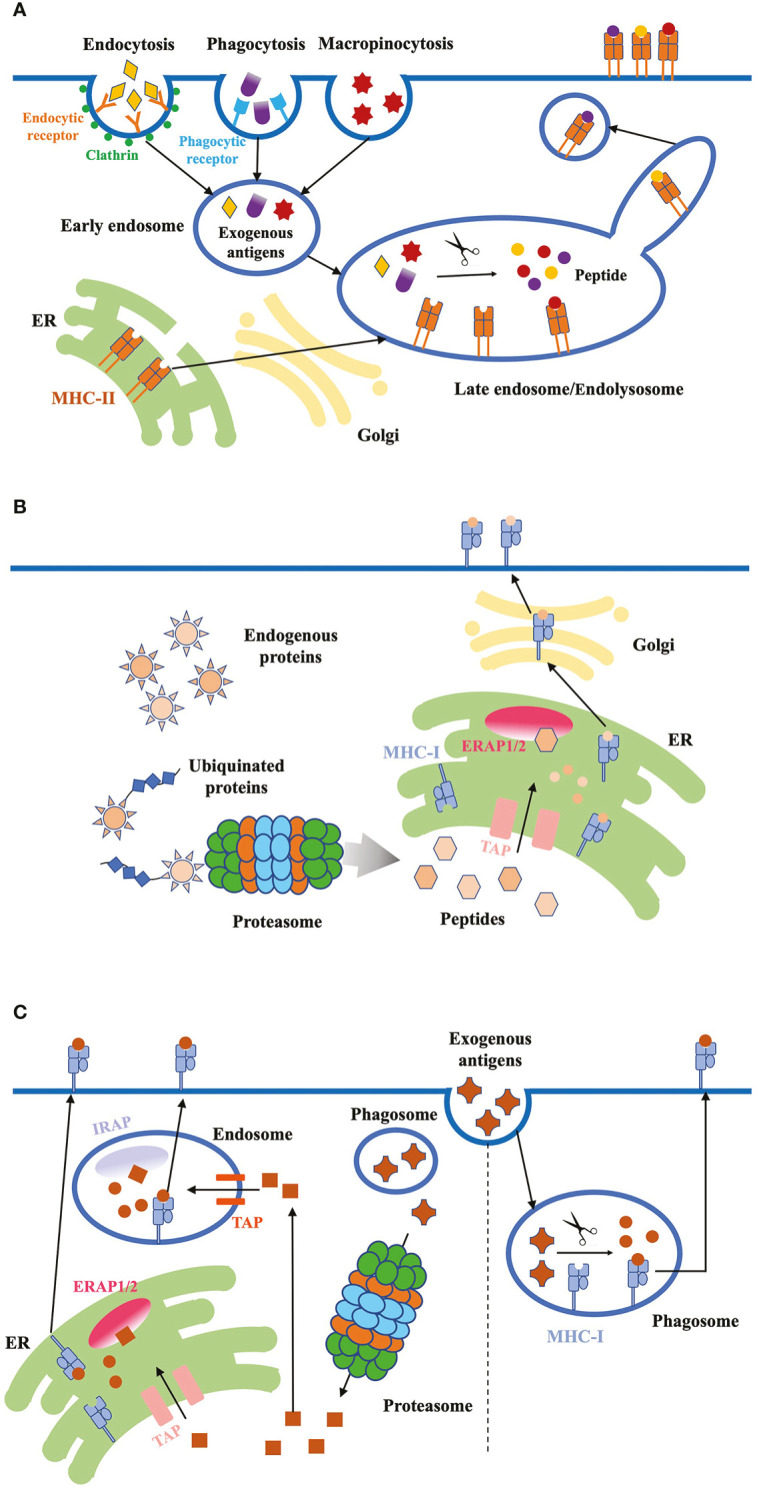
Antigen internalization, processing and presentation. **(A)** Exogenous proteins. DCs use three major ways to internalize exogenous antigens: (1) receptor-mediated endocytosis; (2) phagocytosis; (3) micropinocytosis. Exogenous antigens are delivered from early endosomes to late endosomes/endolysosomes, where they are degraded into peptides. These high-affinity peptides bind to MHC-II molecules, and the peptide-MHC-II complex are transported to the plasma membrane through the tubules and vesicles derived from the endosome. **(B)** Endogenous proteins. Cytosolic proteins first undergo proteolysis, then the peptides are translocated into the ER through TAP proteins. ERAP1/2 in the ER lumen further digests the peptides to fit the MHC-I. Finally, peptide-MHC-I complex are transported to the plasma membrane through classical secretion. **(C)** Antigen cross-presentation. Exogenous antigens can be presented on MHC-I molecules through two pathways: the vacuolar and cytosolic pathway. In the vacuolar pathway, engulfed antigens are digested into peptides in phagosomes, where MHC-I molecules bind to the peptides. In the cytosolic pathway, antigens first enter the cytosol and are degraded into peptides by proteasome. Peptides are transported into endosome or ER through TAP, and further trimmed by IRAP or ERAP, respectively. Trimmed peptides bind to MHC-I and the complex are transported to the plasma membrane.

DCs process both endogenous and exogenous antigen through a series of proteolytic and other enzymatic facilities, including endosome, lysosome and proteasome. Mature DCs increase expression level of MHC-I and MHC-II molecules to load peptides from antigenic proteins in endosome or endoplasmic reticulum (ER). DCs then deliver the peptide-MHC complexes to the plasma membrane, presented the complexes to naïve T cells and initiate the adaptive immune response (Land, 2018) (**Figure 2**).

#### 3.3.1 Exogenous antigen processing

Exogenous antigens are from bacteria and fungi pathogens, dead virus-infected cells and tumor cells. DCs internalize exogenous antigens mainly through three pathways: (1) Receptor-mediated endocytosis: engulfing soluble antigenic molecules in clathrin-coated vesicles which are transported to endosomes (Taylor et al., 2011). (2) Phagocytosis: engulfing receptor-binding insoluble particulate antigens from dead cells or pathogenic organisms into phagosomes. These antigen-loading phagosomes fuse with lysosomes to form phagolysosomes for enzymatic digestion of antigens (Stuart and Ezekowitz, 2005). (3) Macropinocytosis: internalizing non-specific antigens in large endocytic vacuoles independent of specific receptors (Lim and Gleeson, 2011), the main mechanism of virus acquisition.

Internalized exogenous proteins are progressively transferred to early and late endosomes. Endosomes fuse with lysosomes to form endolysosome where the proteins are degraded into peptides. After biosynthesis in the ER, MHC-II molecules are transported to late endosomes where they get functionally mature, and bind to the high-affinity antigenic peptides. Finally, the peptide-MHC-II complexes leave the endosome and are delivered to the cell surface for antigen presentation to CD4^+^ T cells (Roche and Furuta, 2015). The transit process of peptide-MHC-II complex from endosome to the plasma membrane has been studied by multiple imaging techniques with high quality tracking signals. Live-cell imaging revealed that late-endosomes and lysosomes formed tubule-like structures, which could separate from the original sites as vesicles and transport the MHC-II molecules to the plasma membrane. Total internal refection fluorescence microscopy (TIRFM) and spinning disk confocal microscopy further showed that tubular-vesicular structures associated and tethered near the cell surface, and later fused with the membrane (Boes et al., 2002; Chow et al., 2002). Meanwhile, there is another type of phagosomal tubules that don’t fuse with plasma membrane. Instead, phagotubules facilitated content exchange between phagosomes and enhanced the surface expression of peptide-MHC-II complex, which promoted the antigen presentation (Mantegazza et al., 2014).

#### 3.3.2 Endogenous antigen processing

Major endogenous antigenic proteins such as infected virus proteins within DCs undergo proteolysis by the ubiquitin proteasome system (UPS). The peptides in the cytosol are translocated into the ER lumen by transporter associated with antigen processing (TAP) proteins, and are further trimmed by ER aminopeptidases associated with antigen processing (ERAP)1/2 to fit the MHC-I groove. Finally, MHC-I molecules that are also assembled in ER bind the high-affinity peptides, form complex and are transported to the cell surface through the classical secretory pathway. The bound peptides are presented for CD8^+^ T cell recognition (Leone et al., 2013).

#### 3.3.3 Antigen cross-presentation

DCs also load exogenous antigens on MHC-I molecules for cross-presentation to CD8^+^ T cells: the exogenous bacterial and viral antigens, or antigens released from necrotic cells are engulfed by DCs, form complex with MHC-I molecules, activate CD8^+^ T cell and initiate the CTL response. There are two main cross-presentation pathways: the vacuolar and cytosolic pathway. In the vacuolar pathway, internalized antigens are digested into peptides in the endocytic compartments (endosome or phagosome), then the peptides are loaded on MHC-I molecules in the same vesicle. This process is TAP protein independent. In the cytosolic pathway, engulfed exogenous antigens are first transported across the endosomal membrane into the cytosol with an unknown mechanism, and degraded into peptides through proteasome. The peptides are transported either into ER or endosome through TAP, where they are further trimmed to fit the groove of MHC-I molecules. Peptides from soluble antigens are transported into endosomes and trimmed by endosomal insulin-responsive aminopeptidase (IRAP), while peptides from particulate antigens can be trimmed in both ER and endosomes (Embgenbroich and Burgdorf, 2018). Initially, TAP proteins are considered to be necessary for cytosol-to-ER transfer of peptides. However, recent study found alternative pathways to transfer MHC-I to peptides when virus infection blocked TAP proteins. DCs changed the subcellular location of MHC-I molecules to ER-Golgi intermediate compartment, then delivered MHC-I to the phagosomes containing internalized antigens by SNARE protein Sec22b-mediated vesicular traffic. DCs use this noncanonical pathway to rescue antigen cross-presentation when virus blocks TAP (Barbet et al., 2021).

Bacterial antigen processing includes endogenous/exogenous/cross-presentation pathways. Peptides from cytoplasmic bacterial proteins that are digested by proteosome binds to MHC-I. While internalized bacterial proteins are either degraded in endosomes and the peptides bind to MHC-II, or undergo cross-presentation pathway and bind to MHC-I; The viral antigens bind to MHC-1 through two different pathways. If DCs are infected with a virus, the synthesized viral proteins in the cytoplasm undergo endogenous antigen processing. If DCs take up extracellular viral antigens (e.g. antigens from dead virus-infected cells), the antigen are transported to MHC-I for cross-presentation. Fungal and parasite proteins internalized into DCs usually undergo exogenous antigen processing, and the antigenic peptides bind to MHC-II.

In summary, different pathogen invasions induce different DC maturation process through specific antigen recognition and downstream signaling pathways. They impact DC maturation in many aspects, including cytokine expression (interleukin, TNF or IFN), immune effector cell activation (innate immune cell, CD8^+^ and CD4^+^ T cell) and specific immune response induction (CTL or CD4^+^ T_H_ cell response). The specialized DC maturation process targeting each type of pathogen invasion makes great contributions to protect the body from different infections (Summarized in **Table 2**) (Roy and Klein, 2012; Motran et al., 2017; Shepherd and McLaren, 2020; Marongiu et al., 2021).

**Table 2:** DC maturation process induced by different pathogens.

	Bacterium	Virus	Fungus	Parasite
PRRs and downstream pathways	TLRs at the plasma membrane NLRs in the cytoplasm NF-kB and MAPK pathway	TLRs at endosome RLRs in the cytoplasm IRF pathway	TLR2 and CLRs at the plasma membrane NF-kB and pathway	TLR11 and TLR12 at the plasma membrane NF-kB and MAPK pathway
Antigen processing pathway	endogenous/exogenous/cross- presentation pathway MHC-I and MHC-II	endogenous/cross-presentation pathway, MHC-I	Exogenous pathway, MHC-II	Exogenous pathway, MHC-II
Effects on DC maturation and immune response type	DCs secrete pro-inflammatory cytokines (TNF and interleukin); Induce CTL response to kill infected cells; Induce CD4 + TH cell response: provide cytokines to help CD8+ T cells and activate phagocytic cells to kill bacteria.	DCs secrete type I IFN to clear virus; Induce CTL response to kill viral affected cells; Induce CD4 + TH cell response: help CD8+ T cells to destroy infected cells and potentiate the functions of NK cells and macrophages.	DCs secrete pro-inflammatory cytokines; Activate innate effector cells (neutrophils and macrophages); Induce CD4 + TH cell response: generate IFNy (Th1) or IL- 17 (Th17)	DCs secrete pro-inflammatory cytokines (IL-6/12 and TNF-a); Promote Th1 and Th2 response; The interference in the TLR- induced DC maturation

PRR, pattern recognition receptor; TLR, toll-like receptor; NLR, nucleotide oligomerization domain (NOD)-like receptor; RLR, retinoic acid-inducible gene I-like receptor; TNF, tumor necrosis factor; IL, interleukin; CTL, cytotoxic T lymphocyte.

#### 3.3.4 Application of super-resolution microscopy in studying antigen processing

In the past two decades, fast development of super-resolution microscopy techniques overcomes the resolution limit imposed by conventional light microscopy, and greatly expands our knowledge about protein arrangement and cellular structure. When DCs encounter antigenic materials, PRRs on plasma membrane become concentrated at the contacting site. Direct stochastic optical reconstruction microscopy (dSTORM) provided high resolution data on nanoscale spatial rearrangement of C-type lectin receptors in fungal contacts. Membrane receptors significantly increased the nanodomain area at DC-fungus contacting site, which was critical for regulating phagocytic efficiency (Itano et al., 2014). Stimulated emission depletion (STED) microscopy visualized the internalization and trafficking of virus more accurately, showing the temporal shift of the virus particles from early endosomes to late endosomes, and revealing the kinetics of viral protein within DCs (Baharom et al., 2017). STED microscopy also observed internalization of bacterial C3 toxins with other “cargo” proteins into inner lumen of early endosomes, revealing a potential novel way to deliver foreign proteins into DCs (Fellermann et al., 2020). Thus, super-resolution microscopy techniques greatly increase our understanding of membrane protein organization, antigen processing and signal transduction.

### 3.4 Cell morphological change

DCs undergo a remarkable cytoskeletal change and deformation during maturation. Mature DCs appeared an irregular shape with rough surface, which were quite different from the round-shape immature DCs with smooth surface. The rich ruffles on the cell membrane and long protrusions are considered to be the morphologic features of the matured DCs. More subcellular organelles, such as lysosome and ER in the cytoplasm increase cell height and volume. The enlargement of cell surface area favors enhanced expression level of MHC molecules and costimulatory molecules. The change of cell shape also enhanced the adhesion force, promoting cell-cell interaction between DCs and other immune cells (Xing et al., 2011). Cytoskeletal remodeling by actin polymerization in the mature DCs increased cortical stiffness and enhanced efficiency of antigen-presentation and T cell priming (Blumenthal et al., 2020). In summary, mature DCs are well-prepared for antigen presentation and information transfer in molecular and cellular level.

## 4 DC Migration

### 4.1 Cytoskeleton re-organization

Extracellular antigens in the peripheral tissues are internalized by non-motile DCs (Jahnsen et al., 2006; Thornton et al., 2012; Farache et al., 2013), indicating that antigen capture and cell migration are antagonistic processes. Sensing microbial component modifies the dynamics of actin cytoskeleton and location of motor protein. Cdc42-Arp2/3-dependent F-actins at the front of cell promoted antigen uptake but limit migration. This actin nucleation was strongly reduced during DCs maturation, then the predominant F-actin pools were translocated to the cell back and facilitated chemotactic migration. Thus, the transfer of actin nucleation sites promoted the intrinsic migratory capacity of mature DCs (Vargas et al., 2016). Similarly, actin-based motor protein myosin II was associated with both antigen capture and DC migration. Enrichment of myosin II A at the front of immature DCs facilitated antigen delivery to endolysosomes, but reduced the speed of DC locomotion. (Chabaud et al., 2015). When DCs engulfed the microbiol antigens, elevated nuclear translocation of transcription factor EB (TEBB) promoted the expression of transient receptor potential cation channel, mucolipin subfamily member 1 (TRPML1). TRPML1 triggered calcium release from lysosome, activated myosin IIA, accumulated F-actin at the cell back and triggered the fast DC migration. Therefore, the TEBB-TRPML1 axis was essential for altering the migration mode of DC by re-organizing the actin cytoskeleton and motor protein upon antigen sensing and internalization (Bretou et al., 2017). Migration through the tight tissues often requires cell deformation. Owing to the dynamic feature of the actin and microtubule cytoskeletons, the plasma membrane and cytoplasmic organelles are highly flexible. However, cell nucleus with rigid filaments underneath the nuclear membrane makes migration through narrow pores difficult. DCs generated perinuclear actin network nucleated by Arp2/3, disrupted the nuclear lamina, allowed nuclear deformation and facilitated cell passage through the constriction (Thiam et al., 2016).

### 4.2 Peripheral DCs migrate to lymph nodes

Mature DCs from peripheral tissues such as skin, intestine and lung, migrate and transfer through afferent lymphatic vessels to subcapsular sinus (SCS) of lymph node, and then enter the interfollicular region. Confined to the complex environments of peripheral tissues, it is difficulty to acquire high resolution images of migrating DC by *in vivo* live imaging. Therefore, researchers developed *in vitro* experimental setups such as microchannels (Vargas et al., 2014) and three-dimensional (3D) matrix (Lammermann et al., 2009) that mimicked the confined space of peripheral tissues. Researchers also used photoconvertible protein to label endogenous DCs, quantified migrating DCs in the lymph nodes, and calculated their migration kinetics (Tomura et al., 2014). The noninvasive imaging tools such as positron emission tomography/computed tomography (PET/CT) were applied to track the antigen-loaded DC migration in large animals without tissue depth limitation. (Lee et al., 2017). Since the efficiency of DC-based vaccines is highly dependent on the DC movement to the draining lymph nodes after they enter the human body, understanding the molecular mechanisms that underline DC migration is critical for the development of cancer immunotherapy.

Compared with the random movements of immature DCs, mature DCs migrate in a continuous and directional manner, which is largely regulated by chemokine receptor CCR7. Mature DCs upregulate the expression level of CCR7, which recognizes and binds to CC chemokine ligands (CCL) 19 and CCL21 released by lymphatic endothelial cells. The gradient of CCL21 drived the directional DC migration to lymph nodes (Förster et al., 1999; MartIn-Fontecha et al., 2003; Ohl et al., 2004; Weber et al., 2013). Cell tracking in *ex vivo* dermal tissue showed that DCs cannot approach or enter lymphatic vessels without CCR7 (Weber et al., 2013). Early study of DC migration performed on two-dimensional (2D) surface showed that specific integrin-mediated contacts was necessary for DC entry to lymphatic vessels. For example, the adhesion molecules ICAM-1 and VCAM-1 induced in the dermal lymphatic endothelial cells during skin inflammation played key roles in mediating DC transmission to lymph nodes (Johnson et al., 2006). Integrin-deficient DCs cannot overcome tissue barriers like the endothelial layer (2D substrates). In contrast, DCs locomotion in the 3D-extracellular matrix like interstitium can be driven by the protrusive flow of actin polymerization and actomyosin contraction regulated by small GTPase Cdc42. *In situ* live cell imaging together with 3D chemotaxis assays validated that interstitial DC migration was independent of adhesion receptors (Lammermann et al., 2008; Lammermann et al., 2009). Thus, coordination of multiple protrusions that leads to instantaneous entanglement is crucial for DC movement in geometrically complex environments.

When DCs arrive at afferent lymphatic vessels, they continue moving toward lymph nodes. Histo-cytometric analyses of lymph node sections showed that cDC1s moved to deep paracortical T cell zone, while cDC2s stayed at the T cell-B cell border (Gerner et al., 2012). Wild-type cDC1s (CD8α^+^CD103^+^) transmigrated through the afferent-side of SCS floor, formed prominent leading edge, and migrated to the deep T cell zone. In contrast, CCR7^-/-^ DCs don’t exhibited cell polarization, and largely stayed around the SCS regions, indicating that directional migration depended on the expression of CCR7 (Braun et al., 2011; Kitano et al., 2016). To shape the chemokine gradients of CCL19 and CCL21 across the SCS floor, lymphatic endothelial cells localized at SCS ceiling expressed CCRL1 to scavenge chemokines in the sinus lumen, and drove the entry of DCs into the lymph node parenchyma (Ulvmar et al., 2014). These observations together with DC subsets distribution data from histo-cytometric analysis show that migratory cDC1s aim to reach deep T cell zone, while migratory cDC2s disperse throughout the interfollicular zone and paracortex of lymph nodes (Gerner et al., 2012). CCR7 is crucial for regulating peripheral DC migration and their correct localization within lymph nodes. Whether other factors also play a key role in guiding DC migration is not clear. Combination of transcriptome analysis of migrating DC, cell surface protein interactome data and advanced *in vivo* live imaging techniques could help us find more important regulators.

cDC1s are specialized in cross-presenting antigens to CD8^+^ T cells and mediating CTL response, while cDC2s are largely associated with CD4^+^ T cell priming. Segregation of DC subsets guarantees the colocalization of cDC1 with CD8^+^ T cell, and cDC2 with CD4^+^ T cell, respectively. The different positioning of cDCs creates immunologically distinct regions within lymph nodes, which help regulate priming of particular type of T cells in a more efficient way, fine-tunes and tailors the outcome of immune response. The detailed mechanisms are as follows: Multiple competitive chemoattractant gradients within the lymph nodes form different niches that guide cells to migrate and populate in segregated regions (Eisenbarth, 2019). High expression of CCL19 and/or CCL21 in T cell zone recruit cells which express CCR7. In steady state, CD8^+^ and CD4^+^ T cells that express CCR7 are scattered across the T cell zone (Kitano et al., 2016). However, the two lineages of T cells segregate in the early stage of immunization (Eickhoff et al., 2015; Hor et al., 2015). Upregulation of CXCR5 in CD4^+^ T cells promotes them to migrate from deep T cell zone towards follicles which produce CXCL13, the ligand of CXCR5 (Leon et al., 2012; Chen et al., 2015). But expression of CCR7 keeps CD4^+^ T out of the CXCL13^+^ zone, thus they stay in paracortex and interfollicular zone. In addition, T cell-B cell border and interfollicular zone express oxysterol ligands, which recruits CD4^+^ T cells through binding to the receptor Epstein-Barr virus induced gene 2 (EBI2) (Li et al., 2016; Lu et al., 2017). Migratory cDC2s also express high level of CXCR5 and EBI2, which allows the colocalization of cDC2 and CD4^+^ T cells. On the contrary, CD8^+^ T cells still concentrate within the deep T cell zone. Meanwhile, migratory and resident cDC1s express high level of CCR7 but less CXCR5 and EBI2. Thus, cDC1s prefer migrating and localizing in the same site with CD8^+^ T cells. cDC1s also secret enzymes that degrade oxysterols and create an area lack of EBI2 ligands, which further segregate the cDC1 and cDC2 (Lu et al., 2017).

### 4.3 Resident DCs migrate within lymph nodes

Except for the migration of peripheral DCs, the lymph node-resident DCs also undergo trans-nodal repositioning when stimulated by lymph-borne antigen. In the naïve state, majority of resident cDC1s are distributed throughout the T cell zone, while most resident cDC2s localize near lymphatic sinuses (Leal et al., 2021). *In vivo* tracking by multiphoton intravital microscopy showed that resident DCs (both CD8α^+^ cDC1 and CD11b^+^ cDC2) moved from the T cell cortex to the medullary interfollicular regions within minutes after influenza virus arrival from the afferent lymphatics. Resident DCs captured viral antigens, activated CD4^+^ T cells and initiated anti-virus immune response independent of migratory DCs from peripheral tissues (Woodruff et al., 2014). Meanwhile, vaccine adjuvants, bacterial and virus infection could also induce rapid intranodal relocation of resident cDCs from the lymph node periphery into the T cell zone. The cell repositioning was driven by CCR7-mediated chemotaxis. The repositioning of resident cDCs presented draining antigens to T cells and efficiently activated T cells localized in the deep zone (Leal et al., 2021). Consider that peripheral DCs take hours to internalize and process antigens, and then migrate to lymph nodes, resident DCs can induce early T cell activation and initiate adaptive immune response against draining pathogenic antigens without delay. Taken together, the real-time observation of migratory and resident DCs by advanced imaging technologies exhibit the spatial dynamics of different subtypes of DCs and reveal their contributions to the immune response.

## 5 DC-T cell interaction and T cell activation

**Figure 3:**
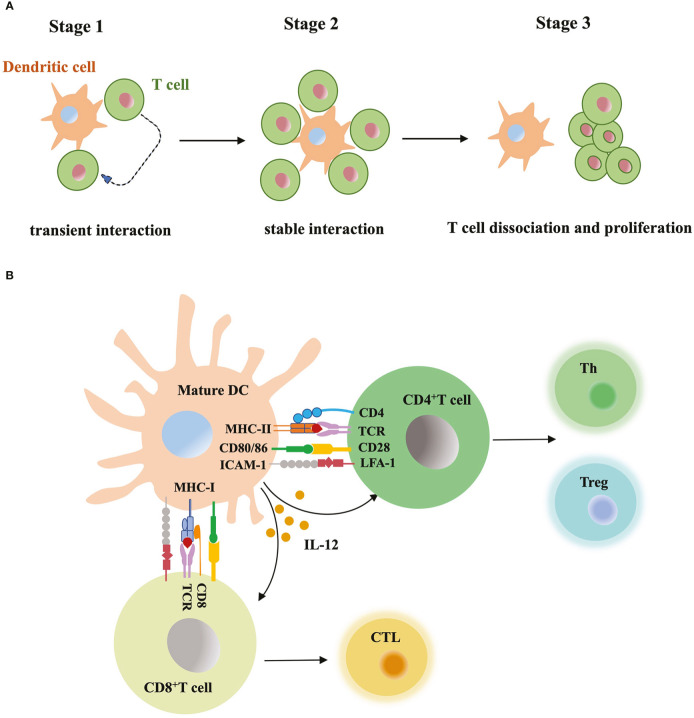
DC-T cell interaction **(A)** Three stages of T-cell priming. (1) Transient interaction; (2) Stable interaction; (3) T-cell dissociation and proliferation. **(B)** T cell activation through DC contact. Mature DCs prime naïve T cells by presenting MHC-bound antigens, providing costimulatory signals (CD80, CD86 etc) and secreting mediators such as IL-12, DCs instruct differentiation of CD8^+^ T cells into cytotoxic T lymphocyte (CTL), while instruct CD4^+^ T cell to differentiate into T helper cell (Th) and regulatory T cell (Treg).

Mature DCs carry antigens and migrate to lymph nodes, where naïve T cells circulate and scan peptide-MHC complex of DCs with T-cell antigen receptors (TCRs) (Stoll et al., 2002). Increased expression level of costimulatory molecules (e.g. CD40, CD80 and CD86) and cytokines secretion from mature DCs promote T cell activation (**Figure 3**). Initial research studying DC-T cell interaction usually relied on static immunohistochemical or fluorescence imaging of tissue sections, or the video taken from *in vitro* model. These data lacked dynamic, high-resolution view obtained from a more complex and physiological *in vivo* model. Now researchers monitor labeled DCs and T cells within intact lymph nodes, trace their behavior and interaction in continuous imaging (Stoll et al., 2002; Bousso and Robey, 2003; Miller et al., 2004). Before immune stimulus, the frequency of T cells is low in the T cell zone, but the high velocity of T cells and mature DCs with rapid shape change enable very efficient cell contacts. The antigen-bearing DC can recruit more than ten T cells at the same time and promotes T cells priming. Activated T cells start to secret cytokines and expand the clone, migrate to inflammation sites or B cell region to aid antibody production. Based on the real-time imaging data, researchers summarized three stages of T-cell priming: (1) T cells undergo stochastic contacts and transient interactions with DCs; (2) T cells decrease their motility and form long-lasting contacts with DCs. At the same stage, T cells start to produce cytokines; (3) T cells dissociate from DCs, migrate with high motility, and proliferate vigorously (Mempel et al., 2004). Short contacts in stage 1 allow T cells to make measurements of antigen dose carried by DCs and set a threshold for subsequent T cell activation. If DCs present large numbers of peptide-MHC complexes, T cells quickly form tight contacts and enter the stage 2. In contrast, if antigen dose is subthreshold, T cells will continue to recirculate and scan (Henrickson et al., 2008). Similarly, T cells expressing low-affinity TCRs require a larger dose of antigens to become activated than those expressing high affinity TCRs (Holler and Kranz, 2003). In stage 3, limiting TCR signaling in T cells and extraction of peptide-MHC complex from the surface of DCs terminate the DC-T cell interaction (Kedl et al., 2002; Schneider et al., 2006). TCR downregulation and block of store-operated calcium entry prevented the recently activated T cells reattaching to DCs. When priming T cells underwent active cell division, they did not contact or form stable interactions with antigen-bearing DCs (Bohineust et al., 2018). DCs-T cell interaction is also regulated by multiple adhesion molecules and GTPase. Intercellular adhesion molecule-1 (ICAM-1) expressed by DCs was essential for long-lasting DC-T cell contacts through binding to LFA-1 in T cells. Without ICAM-1, the survival time of activated CD8^+^ T cell was reduced (Scholer et al., 2008). In addition, GTPase Rac, cdc42 and Ral expressed in DCs maintained the tight and long contacts with T cells, and promote T cell activation (Benvenuti et al., 2004).

DC-T cell contact interface is called immunological synapse made up of three concentric supramolecular activation clusters (SMACs), the central, peripheral and distal SMAC from inside to outside (Basu and Huse, 2017). Central SMAC contains TCR-MHC complex and costimulatory molecular complex, surround by a ring of interacting LFA-1 and ICAM-1 at peripheral SMAC, and transmembrane tyrosine phosphatase CD45 at distal SMAC (Dustin, 2014). DCs form the synapse through polarizing the actin cytoskeleton of cell membrane. The actin remodeling is regulated by Wiskott–Aldrich syndrome protein (WASp) and Rho GTPase Rac. Analysis in the 3D environment of the lymph node showed that WASp was essential to stable the synapse structure and DC-T cell interaction (Benvenuti, 2016). Newly synthesized cytokines like IL-12 are released into the synapse cleft, bind to the receptors and trigger the production of IFN-γ in the T cells. Immunological synapse provides a highly ordered platform for TCR ligation, costimulatory signals transferred and cytokine secretion, which all facilitate T cell activation.

Adaptive immune responses require multiple distinct DC-T cell interactions. During viral infection, initial activation of CD4^+^ and CD8^+^ T cell is spatially separated. In the lymph nodes, infected DCs presented antigens to CD8^+^ T cell and led to T cell proliferation. While CD4^+^ T cells were first activated by non-infected DCs in deeper areas. Later during infection, non-infected CD8α^+^ XCR1^+^ cDCs loaded viral antigens through cross-presentation and presented antigens to both T cell subsets through MHC-1 and MHC-II. This DCs also served as a platform for CD4^+^-CD8^+^ T cell communication and delivered CD4^+^ T cell help to CTL. The cell-location segregation and distinct DC involvement in the initial T cell activation were also observed in the spleen. (Eickhoff et al., 2015). Another study also found spatiotemporally distinct CD-T cell interactions and asynchronous T cell activation after peripheral virus infection. CD4^+^ but not CD8^+^ T cells interacted with migratory DCs carrying viral antigens and got activated. CD8^+^ T cells remained naïve in the early infection stage until lymph node-resident XCR1^+^ DCs received licensing signals from activated CD4^+^ T cells (Hor et al., 2015). Both of these studies highlight the key role of XCR1^+^ DCs as the central platform for CTL activation through the delivery of CD4+ T cell help.

## 6 Conclusion remarks

In this review, we summarized the DC maturation process after infection, including antigen sampling, processing and presentation, DC migration, DC-T cell interaction and T cell activation, and the molecular mechanisms underlying signal transduction and information transfer between immune cells. In addition, the rapid development of optical technologies makes great contribution to our understanding of the DC behavior and function during infection. The representative technique, live animal imaging through intravital multiphoton-microscopy coupled with novel fluorescent labeling have shed new lights into the spatiotemporal relationships between DCs and pathogens, DCs and T cells in a single-cell resolution. While our understanding of DC maturation is more precise, many observations are still heterogeneous, depending on the experimental systems, the origin of the DC population, and the type of stimuli used. Questions arising from these observations are also waiting for the answer, e.g. What happens during long-distance DC migration from peripheral tissues to lymphoid organs *in vivo*; How to distinguish different DC-T cell interactions based on the types of immune stimuli; What is the impact of dynamic, transient DC-T cell interaction on T cell priming, etc. These remaining questions are challenging, and require more sophisticated imaging modalities and lineage tracing methods. We also need to explore the relevance of these finding in human immune system to apply the basic research of DC biology in vaccines development, and medical treatment for infection, cancer and autoimmune disease.

## Author contributions

QX: conceptualization, writing-original draft preparation, review and editing. YX: writing- review and editing. All authors contributed to the article and approved the submitted version.
